# HCFNet: A SAM2-Based Hierarchical Cross-Branch Frequency-Aware Network for Industrial Surface Defect Segmentation

**DOI:** 10.3390/s26144597

**Published:** 2026-07-20

**Authors:** Jiwei Yu, Kecheng Zhou, Ting Wang, Hongxiao Gan, Yu Wang, Shuzhi Gao

**Affiliations:** 1 Institute of Equipment Reliability, Shenyang University of Chemical Technology, Shenyang 110142, China; 2024320@stu.syuct.edu.cn (J.Y.);; 2 Shenyang Institute of Automation, Chinese Academy of Sciences, Shenyang 110016, China; 3 Liaoning Key Laboratory of Intelligent Technology for Chemical Process Industry, Shenyang University of Chemical Technology, Shenyang 110142, China; 4 The State Key Laboratory of Robotics and Intelligent Systems, Shenyang Institute of Automation, Chinese Academy of Sciences, Shenyang 110016, China; zhoukecheng@sia.cn (K.Z.);

**Keywords:** surface defect segmentation, Segment Anything Model, foundation model

## Abstract

Foundation models such as the Segment Anything Model 2 (SAM2) have demonstrated strong performance in image segmentation; however, their application to industrial defect detection faces significant challenges due to the substantial domain gap between natural and industrial images, insufficient sensitivity to fine-grained high-frequency structures, and reliance on manual prompts. To address these issues, this study proposes a Hierarchical Cross-Branch Frequency-Aware Network (HCFNet) to adapt SAM2 for prompt-free industrial defect segmentation. First, a Gated Adapter is introduced into the frozen SAM2 encoder, enabling efficient cross-domain transfer without massive parameter retraining, thereby effectively preserving the pre-trained visual priors. Secondly, a Laplacian-enhanced Auxiliary Branch is designed to explicitly amplify high-frequency components, compensating for the inherent perception limitations of the Transformer backbone and significantly awakening the model’s sensitivity to subtle defects like micro-cracks. Finally, a Cross-branch Multi-scale Fusion Module is proposed to seamlessly align and integrate global semantic information with local structural details in a unified manner, resolving heterogeneous feature distribution conflicts. Extensive experiments on the MVTec AD and VisA datasets demonstrate that the proposed method consistently outperforms SAM2-based baselines in terms of mIoU and mDice. This study establishes an effective approach for leveraging foundation models in automated industrial inspection and is expected to drive advancements in precise defect perception technologies.

## 1. Introduction

Foundation models, particularly the Segment Anything Model (SAM) and its subsequent variants [1,2,3], have significantly propelled the advancement of open-world image segmentation, driven by large-scale pre-training and robust zero-shot generalization capabilities. However, despite their remarkable achievements in natural scene understanding, directly migrating these models to vision-based automated defect detection in industrial scenarios [4] remains a fundamental challenge. This limitation primarily stems from a structural mismatch between the learned representations and the intrinsic properties of industrial data, manifesting in three key aspects:

First, a significant domain shift exists between natural images and domain-specific images (e.g., industrial scenarios), leading to misaligned feature distributions and consequent degradation in segmentation performance [5,6]. Second, although the Transformer-based backbone excels at capturing global semantic information, its inherent architecture lacks explicit mechanisms for modeling high-frequency local details (e.g., micro-cracks and fine scratches), resulting in a “global-strong, local-weak” representation bias. Third, as a prompt-driven framework, SAM2 relies heavily on external manual prompts, which severely limits its applicability in fully automated industrial inspection scenarios [7].

Existing deep learning-based segmentation methods are predominantly tailored to specific tasks [8,9], relying heavily on large-scale annotated datasets and exhibiting limited generalization across diverse industrial environments. Recent efforts to adapt foundation models have primarily focused on Parameter-Efficient Fine-Tuning (PEFT) strategies, such as LoRA [10] and standard Adapters [11]. Although these methods partially alleviate the domain shift, their rigid low-rank transformations lack the capacity for dynamic regulation of adaptation strength, making them prone to interfering with pre-trained knowledge. More critically, these approaches overlook the structural representation gap: merely fine-tuning the Transformer backbone is insufficient to effectively enhance the model’s sensitivity to fine-grained defect boundaries. Conversely, simply introducing additional convolutional branches, devoid of effective alignment mechanisms, often leads to the fragmentation of heterogeneous feature distributions.

To address the aforementioned challenges, we propose a Hierarchical Cross-Branch Frequency-Aware Network (HCFNet) built upon the pre-trained SAM2 model. Instead of forcing a single backbone to simultaneously capture global semantics and local structures, our framework explicitly constructs complementary representations through a cross-branch design and achieves efficient feature integration via a dedicated alignment mechanism.

Specifically, to establish a fully automated, prompt-free segmentation pipeline, we directly adapt the SAM2 encoder by stripping away its prompt dependency. To mitigate the domain shift while preserving pre-trained knowledge, we introduce a Gated Adapter into the frozen SAM2 encoder, enabling parameter-efficient fine-tuning through the dynamic regulation of adaptation signals. To compensate for the Transformer backbone’s deficiency in modeling fine-grained details, we design an auxiliary branch dedicated to local feature extraction. This branch incorporates a Laplacian convolution-based multi-frequency channel attention mechanism, designed to explicitly amplify high-frequency components, thereby heightening the model’s sensitivity to subtle defects such as micro-cracks and tiny dents. Furthermore, to seamlessly integrate the heterogeneous representations from the backbone and the auxiliary branch, we develop a cross-branch multi-scale feature fusion module. By performing channel alignment and resolution-aware feature aggregation, it ensures the consistency and complementarity of feature representations across different scales.

Extensive experiments conducted on two mainstream industrial defect datasets, MVTec AD and VisA [12], demonstrate that our method comprehensively outperforms state-of-the-art approaches in terms of mIoU and mDice metrics. Notably, HCFNet exhibits robust performance gains across different datasets, thoroughly validating its efficacy in bridging the domain shift and enhancing fine-grained defect representations.

The main contributions of this paper are summarized as follows:

(1) We propose a Hierarchical Cross-Branch Frequency-Aware Network (HCFNet) for adapting foundation models to industrial defect segmentation tasks, effectively leveraging the pre-trained knowledge of SAM2 while eliminating the reliance on manual prompts, thereby addressing the critical challenges of domain shift and structural representation limitations;
(2) We design a Gated Adapter for parameter-efficient fine-tuning, which dynamically regulates domain adaptation signals while safeguarding pre-trained knowledge;
(3) We construct a Laplacian convolution-enhanced dual-branch framework, where a Transformer-based backbone branch is complemented by an auxiliary branch for high-frequency feature modeling. Together with a cross-branch multi-scale fusion strategy, the proposed method enables precise modeling of fine-grained details and robust alignment of heterogeneous representations.

The remainder of this paper is organized as follows. Section 2 briefly reviews the related work on industrial defect detection and the development of SAM-based models. Section 3 details the proposed HCFNet framework, systematically introducing the Gated Adapter, the Laplacian-enhanced auxiliary branch, and the cross-branch multi-scale feature fusion module. Section 4 presents the experimental setup and provides comprehensive quantitative and qualitative comparisons with state-of-the-art methods on the MVTec AD and VisA datasets, followed by an ablation study to validate the effectiveness of each core component. Finally, Section 5 concludes this work and discusses potential directions for future research.

## 2. Related Work

### 2.1. Industrial Defect Detection

The core objective of industrial defect detection is to achieve accurate localization and segmentation of abnormal regions in products during the production process. Its technological evolution can be divided into two major stages: traditional methods and deep learning-driven methods. Before the popularization of deep learning, traditional defect detection methods mainly relied on template matching, threshold segmentation, and manually designed feature extractors (e.g., Local Binary Patterns (LBP), Gabor filters, Scale-Invariant Feature Transform (SIFT), etc.). Although these methods are simple to implement and low in deployment cost, they heavily depend on manually designed features based on empirical knowledge, and exhibit extremely poor robustness to illumination variations, product pose differences, and tiny defects, making them difficult to adapt to diverse industrial production scenarios [13,14,15,16].

With the advancement of deep learning technology, data-driven defect detection methods have gradually become mainstream. These methods can be categorized into three types according to the supervision mode: fully supervised, unsupervised, and weakly supervised methods. Fully supervised methods (e.g., FCN-based segmentation models [17], ResNet+FPN-based detection frameworks [18]) require a large number of defect samples with pixel-level annotations. While they can achieve high-precision segmentation, the scarcity of defect samples and extremely high annotation costs in industrial scenarios have greatly limited their practical application scope. To address the challenge of data annotation, unsupervised/weakly supervised methods have emerged as a research hotspot. These methods only require normal samples for training and identify anomalies by modeling the distribution differences in normal patterns. Existing unsupervised algorithms are mainly classified into three categories [19,20,21]: reconstruction-based [22], embedding-based [23,24], and synthesizing-based methods [25,26].

Despite the certain achievements of existing methods in specific scenarios, they still have some limitations: first, the generalization ability is limited—most methods are designed for a single product category or specific defect type, resulting in poor transfer performance across different products and defect types; second, the detail segmentation accuracy is insufficient—they show weak capabilities in identifying and segmenting low-contrast, small-scale defects such as tiny scratches and fine cracks; third, the model adaptation cost is high—for new industrial scenarios, it is often necessary to redesign the network structure or adjust a large number of training parameters [27,28]. These limitations have prompted researchers to explore the introduction of general visual prior knowledge to improve the generalization and detail perception capabilities of models, and the emergence of general-purpose vision foundation models has provided new possibilities for this exploration.

### 2.2. SAM and Applications

To overcome these limitations, general visual foundation models with strong generalization capability have emerged as a promising solution. With the advancement of deep learning, recent research has increasingly focused on large-scale pre-trained models. Among these, the Segment Anything Model (SAM) [1], proposed by Meta, leverages an innovative prompt-driven mechanism and a massive dataset of pre-trained image-mask pairs. This enables SAM to achieve zero-shot/few-shot segmentation in open-world scenarios, thereby breaking the reliance of traditional segmentation models on specific tasks and datasets. This strong generalization ability also makes SAM potentially applicable to a wide range of industrial inspection scenarios, including diverse materials such as ceramics, carbon fiber composites, and steel surfaces.

The core advantage of SAM lies in its strong generalization capability—it can respond to multiple prompt signals such as points, boxes, and text to segment unseen objects and scenes, making it highly adaptable to diverse downstream tasks.

In the field of image segmentation, researchers have carried out extensive adaptation studies based on SAM. For instance, SAM-Med2D [29] improves the segmentation accuracy of organ boundaries and lesion regions by fine-tuning the decoder of SAM and incorporating anatomical priors of medical images; the SAM-Adapter series [30,31] applies SAM-based models to camouflaged object segmentation, shadow detection, and medical image segmentation by introducing Adapter technology, providing standardized benchmarks for research in this field; the SAM2-Unet series [10,32] adopts the image encoder of the SAM combined with a Unet decoder for downstream task adaptation, offering a lightweight solution for foundation model applications; SAM2-UNext [33] fuses the image encoders of Dinov2 and SAM2 via a fusion module and verifies the superiority of its method on multiple tasks. Jmal et al. [34] proposed an innovative method combining the Segment Anything Model (SAM) with multi-stage refinement optimization to address the insufficient segmentation accuracy of olive trees in satellite images; Rafaeli et al. [35] proposed two Prompt generation methods to guide the SAM model for sinkhole image segmentation. These studies have verified the potential of SAM as a general encoder, but most existing SAM-based research focuses on fields such as medical image segmentation, camouflaged object detection, and remote sensing images [36,37], while research on applying SAM to industrial defect detection remains scarce.

Given its strong generalization capability, SAM has the potential to be applied across diverse industrial inspection scenarios (e.g., ceramics, carbon fiber materials, and steel plates). However, currently, most studies applying SAM to industrial defect detection focus on adaptation strategies. For example, Ref. [38] proposed using Unet for preliminary segmentation to generate prompts that guide the SAM for tool wear segmentation; DA-SAM [39] enables SAM to adapt to industrial segmentation tasks by introducing LoRA-based efficient fine-tuning. KairosAD [40] utilizes SAM as a feature extractor, with an extremely lightweight fully connected anomaly scoring head attached for industrial image anomaly detection. Although the aforementioned methods have achieved certain results, they have not fully addressed the issues of domain shift and local detail modeling at the encoder level.

Since the encoders of the SAM series are pre-trained on natural images, the domain shift problem limits their feature representation capability in industrial scenarios. Furthermore, although the ViT [41] architecture adopted by SAM excels at capturing long-range dependencies via its global self-attention mechanism, it lacks an explicit local receptive field design, making it difficult to effectively model the tiny details and fine boundaries commonly found in industrial defects [42]. Therefore, how to achieve domain adaptation of the SAM encoder through parameter-efficient fine-tuning strategies while supplementing local detail modeling capability has become the key to improving its application effectiveness in industrial defect detection.

## 3. Method

### 3.1. Hierarchical Cross-Branch Frequency-Aware Network

**Figure 1:**
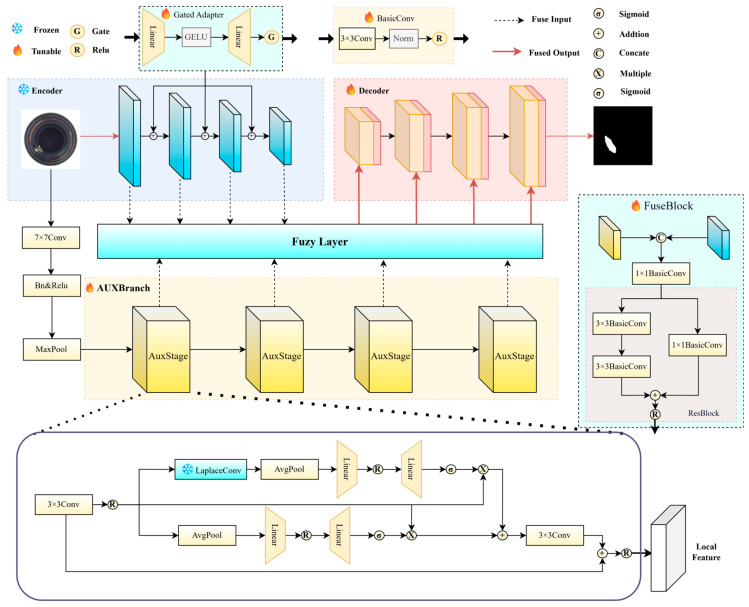
Overview of the proposed HCFNet.

To address the aforementioned issues, we propose a Hierarchical Cross-Branch Frequency-Aware Network (HCFNet) for industrial defect segmentation. The proposed framework is built upon the pre-trained weights of SAM2, as illustrated in Figure 1, and consists of several core components, including an encoder, a decoder, parameter-efficient adapters, a feature fusion module, and an auxiliary branch module. Herein, we adopt the architecture of SAM2-UNet, which has achieved excellent performance across multiple tasks and established itself as one of the benchmark models for comparison in SAM-based image segmentation tasks [32,43,44,45]. While leveraging the pre-trained weights of SAM2, we discard several task-irrelevant components in the original model, including the memory attention mechanism, prompt encoder, memory encoder, and memory bank, as they are not essential for image segmentation in our prompt-free industrial setting.

Compared with traditional U-Net-based segmentation models, which typically train from scratch and require large-scale annotated data to reach competitive accuracy, the proposed method adopts the Hiera image encoder of SAM2 as its backbone. The hierarchical pretrained representation provides strong, general visual priors that substantially reduce the data dependence of downstream defect segmentation, making the model particularly effective under limited-data conditions [46]. Moreover, the multi-scale feature hierarchy of Hiera naturally aligns with defects of varying sizes, whereas a plain U-Net encoder must learn such multi-scale representations from scratch. We retain the U-Net-style decoder and deep supervision strategy, and further enhance the original encoder with a parameter-efficient adaptation mechanism, thereby combining the data efficiency of a pretrained foundation backbone with the localization capability of a U-Net decoder. More importantly, three key components are newly introduced: (1) a Gated Adapter-based parameter-efficient fine-tuning module for effective domain adaptation; (2) a dual-branch architecture to jointly model global semantic information and local fine-grained details in a complementary manner; and (3) a cross-branch feature alignment and multi-scale fusion mechanism to enhance heterogeneous feature interaction and representation consistency.

This overall architecture provides a unified framework for industrial defect segmentation by jointly modeling global semantic information and local fine-grained structures in a complementary manner. To fully exploit the pre-trained knowledge of SAM2 while bridging the domain gap and maintaining computational efficiency, the proposed network is further refined through several specialized components. In the following subsections, we describe these components in detail.

### 3.2. Gated Adapter for SAM2 Image Encoder

The main Image Encoder proposed in this paper is built upon the pre-trained Hiera encoder of SAM2 [47], with Adapter modules inserted into it to enable Parameter-Efficient Fine-Tuning (PEFT). Unlike the basic ViT [40] encoder, Hiera features a hierarchical network structure that can output feature maps at 4 different levels. For Hiera-L, the number of channels corresponding to each level is given by $C_{i}\in \{144,\;288,\;576,\;1152\},i=1,2,3,4$, enabling effective modeling of multi-level visual semantics.

To enable parameter-efficient adaptation, we insert Adapter modules into each stage of the Hiera encoder. During training, the original SAM2 backbone is kept frozen, and only the inserted Adapter modules are optimized. This follows the paradigm of Parameter-Efficient Fine-Tuning (PEFT), which significantly reduces trainable parameters while preserving the pre-trained knowledge.

**Figure 2:**
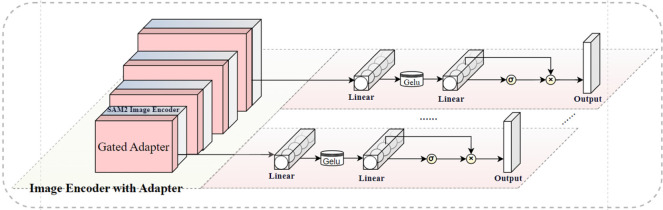
Image Encoder and GatedAdapter architecture.

Gating mechanisms have been widely adopted in large-scale foundation models to adaptively regulate information flow and suppress irrelevant responses. Their effectiveness has been well demonstrated in large language models [48], suggesting that similar dynamic control may also be beneficial for vision foundation models. Building upon this observation, we designed a Gated Adapter as the core adaptation unit within each inserted Adapter module. The overall architecture is illustrated in Figure 2. The Gated Adapter adopts a bilinear transformation structure combined with a learnable gating mechanism.

First, a downsampling linear layer is employed to reduce the feature dimensionality and aggregate relevant information. This is followed by the application of a GELU activation function to facilitate non-linear feature transformation. Subsequently, an upsampling linear layer restores the feature dimensions to the target scale. Finally, a gating unit is introduced to adaptively reweight feature responses along channel or spatial dimensions, thereby suppressing irrelevant information and enhancing discriminative representations.

The above process can be formulated as:

(1) $$Z=\sigma (W_{g}X)⊙W_{u}\;GELU\left(W_{d}X\right)$$

where X denotes the input feature map, $W_{d}$ and $W_{u}$ are the downsampling and upsampling linear layers, respectively, $W_{g}$ denotes the learnable gating projection, and σ(⋅) denotes the gating operation, and ⊙ represents element-wise multiplication.

By explicitly inserting Adapter modules into the hierarchical encoder, the model achieves efficient domain adaptation while maintaining the strong generalization capability of SAM2. However, although the Gated Adapter enhances global semantic modeling, the Transformer-based backbone still lacks sufficient local detail perception capability (e.g., micro-cracks). To address this limitation, we introduce an auxiliary branch for local detail enhancement in the following section.

### 3.3. Auxiliary Branch for Local Detail Enhancement

**Figure 3:**
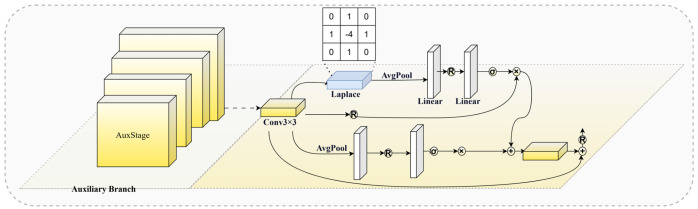
Auxiliary Branch Module for Supplementing Local Detail Information of the Model.

To compensate for the limited local detail perception capability of the Transformer-based backbone, we design a multi-stage auxiliary branch to extract fine-grained defect features in a complementary manner. As illustrated in Figure 3, the auxiliary branch is aligned one-to-one with the four hierarchical stages of the Hiera backbone and consists of four AuxStage sub-modules. During training, supervision is directly applied to the raw images, enabling the branch to learn low-level detailed representations more effectively. By combining shallow feature extraction with complementary enhancement of local structures, the proposed auxiliary branch improves the model’s suitability for industrial surface defect segmentation.

The transformation process of each AuxStage can be concisely formulated as:

(2) $$F_{out}^{l}=\sigma (BN(W_{3\times 3}^{\left(2\right)}*(F_{freq}^{l}+F_{se}^{l})))+F_{1}^{L}$$

where $F_{1}^{L}=\sigma (BN(W_{3\times 3}^{\left(1\right)}*X^{l}))$, $F_{freq}^{l}$ and $F_{se}^{l}$ denote the frequency-enhanced attention and channel attention branches, respectively.

The frequency-enhanced branch is defined as:

(3) $$F_{freq}^{l}=F_{1}^{L}⊙\sigma (MLP(GAP(W_{lap}*F_{1}^{L})))$$

Here, $W_{lap}$ denotes the Laplacian convolution kernel, which enhances high-frequency details. This design explicitly enhances high-frequency components, which are crucial for accurately capturing subtle defect patterns such as edges and micro-structures, thereby improving the model’s sensitivity to fine-grained anomalies in industrial scenarios.

The detailed transformation process of each AuxStage sub-module is described as follows. The input data first passes through a 3×3 convolutional layer to complete the preliminary extraction of shallow features and spatial dimension alignment. A multi-frequency channel attention mechanism based on Laplacian convolution is introduced to enhance the perception of high-frequency details (e.g., crack edges and tiny dents). Then, another 3×3 convolutional layer is employed for feature integration and dimension calibration. Meanwhile, a residual connection is embedded to facilitate gradient propagation and stabilize training.

Ultimately, the features extracted from each level of the auxiliary branch are adaptively fused with the corresponding backbone features via a lightweight fusion module, achieving complementary enhancement of local detailed features and global semantic information.

By jointly modeling frequency-aware and channel-wise attention, the proposed auxiliary branch effectively captures both high-frequency details and global contextual dependencies, thereby compensating for the inherent limitations of the Transformer-based backbone in modeling fine-grained features.

By introducing an auxiliary branch to complement the limitations of the backbone encoder, the model is able to capture fine-grained local details while preserving strong global semantic representations, thereby achieving more comprehensive feature modeling. However, due to the inherent differences in feature distributions and representation scales between the two branches, effective fusion of heterogeneous features remains challenging. To address this issue, we propose a Cross-Branch Multi-Scale Feature Fusion module in Section 3.4 to enable effective integration of the complementary representations.

### 3.4. Cross-Branch Multi-Scale Feature Fusion

**Figure 4:**
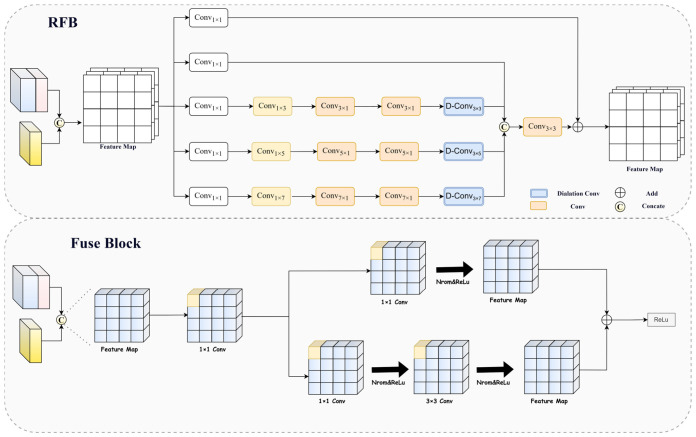
Feature Fusion Modules for Fusing Dual-Branch Feature Information.

In this section, we design a cross-branch feature fusion module to integrate representations from the auxiliary branch and the backbone encoder. The proposed module performs channel alignment and feature refinement to effectively combine global semantic information with local structural details. The overall architecture is illustrated in Figure 4.

As shown in Figure 4, the fusion framework consists of two components applied at different network depths. The Refined Feature Block (RFB), adopted from SAM2-UNet, is employed in the upper layers (Layers 3 and 4) to enhance large-scale defect perception. To achieve a balance between representation capability and computational efficiency, we adopt a hierarchical design: RFB is used in the upper layers, while the proposed FuseBlock is applied in the lower layers (Layers 1 and 2) for efficient feature refinement.

The fusion process can be formulated as:

First, a channel-wise concatenation is performed between the backbone and auxiliary features:

(4) $$F_{c}^{l}=\sigma (BN(W_{1\times 1}*Concat(F_{b}^{l},F_{a}^{l})))$$

The fused feature is then refined via a residual convolutional block:

(5) $$F_{out}^{l}=F_{c}^{l}+R(F_{c}^{l})$$

where $F_{b}^{l}$ and $F_{a}^{l}$ denote the backbone and auxiliary features at level l. $W_{1\times 1}$ is used for channel alignment, and  R(·) represents the residual feature refinement module composed of convolutional layers.

This design effectively integrates complementary information from both branches, enhancing local detail preservation while maintaining global semantic consistency across different scales.

Furthermore, a hierarchical assignment strategy is adopted for different network depths:

(6) $$F^{l}=\left\{\begin{array}{c} \mathrm{RFB}(F_{c}^{l}),l\in \{3,4\}\\ \mathrm{FuseBlock}(F_{c}^{l}),l\in \{1,2\} \end{array}\right.$$

This resolution-aware design achieves a better trade-off between accuracy and computational efficiency in industrial defect segmentation.

Finally, the fused features are fed into a U-Net-style decoder to progressively reconstruct high-resolution segmentation masks under a deep supervision strategy. With the enriched multi-scale feature representations generated by the fusion module, the decoder iteratively reconstructs the spatial resolution, ensuring robust convergence through deep supervision, as detailed in the following section.

### 3.5. Decoder and Training Strategy

In this work, we adopt the SAM2-UNet architecture [42] for the decoder, following a classical U-Net design composed of three cascaded decoder blocks. Each block consists of two sequential Conv–BN–ReLU layers, which progressively refine feature representations during the upsampling process.

At each decoding stage, a segmentation head is applied to generate intermediate segmentation results $S_{i},\;i\in \{1,2,3\}$. To ensure effective training and improve convergence stability, we apply a deep supervision strategy, where all intermediate outputs are supervised during training.

The overall loss function combines the weighted intersection over union (IoU) loss and weighted Binary Cross-Entropy (BCE) loss at each decoding level. Specifically, the total loss function is formulated as:

(7) $$L_{total}=\sum_{i=1}^{3}\left(\lambda_{1}L_{wIoU}(G,S_{i})+\lambda_{2}L_{wBCE}(G,S_{i})\right)$$

where G denotes the ground truth, and $S_{i}$ represents the predicted segmentation output at the i-th decoder level. The parameters $\lambda_{1}$ and $\lambda_{2}$ are weight coefficients controlling the contributions of IoU and BCE losses, respectively. In practice, we fine-tune these hyperparameters during training to achieve an optimal trade-off between these two loss terms.

## 4. Experiments

### 4.1. Experimental Settings

#### 4.1.1. Experimental Details

To comprehensively evaluate the effectiveness and generalization capability of the proposed method across diverse industrial contexts with varying material properties and defect characteristics, extensive comparison and ablation experiments were conducted on two representative industrial anomaly detection datasets, MVTec AD and VisA. The evaluation metrics include commonly used segmentation indicators, such as mIoU, mDice and AUROC. The SAM2 variant employed in this study is sam2.1_hiera_large.pt. The model is optimized using AdamW with an initial learning rate of 1 × 10^−3^ and weight decay of 5 × 10^−4^, scheduled by cosine annealing (minimum learning rate 1 × 10^−7^), and trained for 30 epochs at a batch size of 12 with an input resolution of 352 × 352. Only the parameter-efficient modules (Gated Adapters, auxiliary branch, fusion modules, and decoder) are updated, amounting to 28.4 M trainable parameters (11.8% of the 240.5 M total), while the SAM2 Hiera-L backbone remains frozen. All experiments are conducted on a single NVIDIA RTX 3090 GPU. Both datasets are partitioned using a consistent split ratio (70% training, 15% validation, 15% testing). We note that this study addresses supervised defect segmentation with pixel-level mask annotations under this split, which is a segmentation setting rather than the one-class unsupervised anomaly-detection protocol commonly associated with MVTec AD and VisA; the reported metrics therefore reflect supervised segmentation accuracy. All input images are converted to RGB and uniformly resized to 352 × 352 (ground-truth masks resized with bicubic interpolation), and pixel values are normalized using the ImageNet mean (0.485, 0.456, 0.406) and standard deviation (0.229, 0.224, 0.225).

#### 4.1.2. Evaluation Metrics

Let P denote the predicted probability/segmentation mask and G the ground-truth binary mask. The mean Dice coefficient (mDice) measures region overlap and is defined as:

(8) $$mDice=\frac{2|P\cap G|}{\left|P|+|G\right|}.$$

The mean Intersection over Union (mIoU) is defined as:

(9) $$mIoU=\frac{\left|P\cap G\right|}{\left|P\cup G\right|},$$

where |P∪G|=|P|+|G|−|P∩G|. The Area Under the Receiver Operating Characteristic curve (AUROC) is obtained by thresholding the predicted probability map at multiple levels and integrating the true-positive rate against the false-positive rate, evaluating pixel-level anomaly separability. The mean Absolute Error (MAE) averages the per-pixel absolute difference:

(10) $$MAE=\frac{1}{N}\sum_{i=1}^{N}|P_{i}-G_{i}|.$$

All metrics are first computed per image and then averaged over the test set.

### 4.2. Datasets Overview

**Figure 5:**
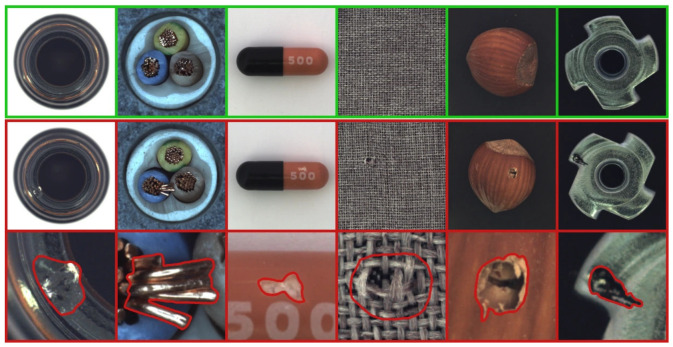
Sample images from the MVTec AD dataset.

Two representative industrial anomaly detection benchmark datasets, MVTec AD [4] and VisA [12], are selected for this experiment to comprehensively validate the effectiveness and generalization ability of the proposed method. Detailed information on both datasets is as follows:

MVTec AD [4] is one of the most authoritative and widely used benchmark datasets in the field of industrial anomaly detection and anomaly segmentation. It contains 15 categories and 73 anomaly types, with sample images shown in Figure 5. For this experiment, only the data with pixel-level annotations are used, consisting of 1260 data samples in total. The dataset is split into 70% for training, 15% for validation, and 15% for testing, resulting in 880 samples for training, 190 for validation, and 190 for testing. This dataset covers a variety of industrial materials and morphologies, including representative categories such as ceramic surfaces (e.g., tiles), metal components, and texture materials, with a diverse range of defects such as scratches, holes, and cracks.

**Figure 6:**
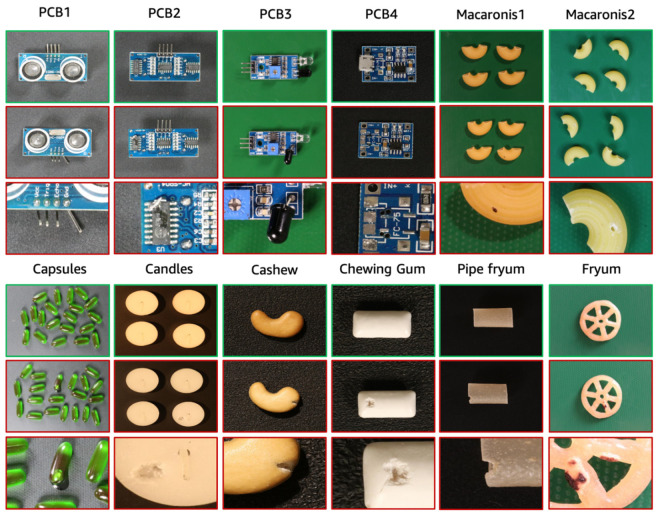
Sample images from the VisA dataset.

VisA [12] is another industrial anomaly detection dataset focused on defect detection tasks in real industrial production scenarios. It covers a wide variety of industrial product types, and its defect types are closely aligned with the complex conditions encountered in actual production, with sample images shown in Figure 6. The data exhibits high diversity and complexity, with challenging defect types. In this experiment, pixel-level annotated samples are used for training and testing, and the dataset is split in the same ratio as MVTec AD (70% for training, 15% for validation, and 15% for testing), ensuring fairness in the experimental comparison. The split data samples are used to verify the generalization ability of the proposed method, compensating for the limitations of experiments on a single dataset.

### 4.3. Comparison with State-of-the-Art Methods

#### 4.3.1. Quantitative Results

To comprehensively evaluate the effectiveness of the proposed method, we conduct comparative experiments on two representative industrial anomaly detection datasets, MVTec AD and VisA. The evaluated methods include SAM-based models (SAM2-UNet, SAM2-UNext, SAM-Adapter) and traditional baselines (U-Net with different epochs).

**Figure 7:**
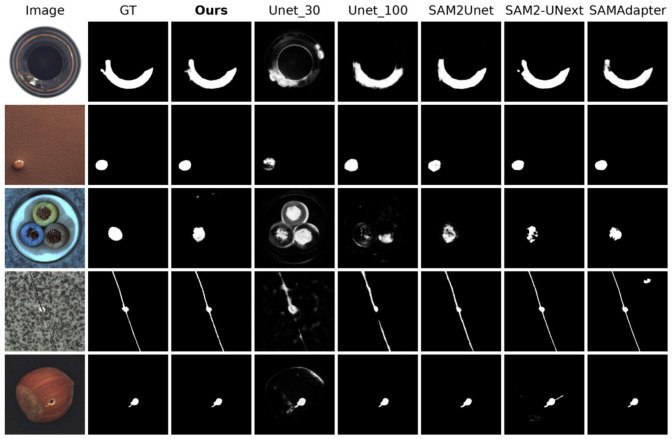
Visualization results on the MVTec AD dataset.

**Figure 8:**
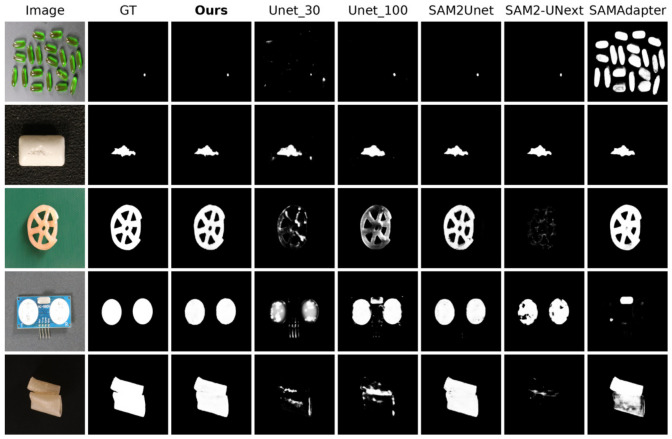
Visualization results on the VisA dataset.

Visual comparisons on representative defect categories are shown in Figure 7 and Figure 8 for MVTec AD and VisA, respectively. The quantitative results are reported in Table 1.

#### 4.3.2. Analysis of Experimental Results

From the results in Table 1, the proposed method achieves strong performance on both datasets. On MVTec AD, it obtains the best mDice (0.802) and mIoU (0.695), outperforming SAM2-UNet by 0.031 and 0.038, respectively. It also achieves the lowest MAE (0.007), indicating improved pixel-level localization accuracy.

On the VisA dataset, the proposed method maintains its advantage, achieving mDice and mIoU scores of 0.645 and 0.518, respectively, with consistent improvements over SAM2-UNet. Meanwhile, it achieves the lowest MAE (0.004), further demonstrating accurate defect localization under complex backgrounds. The AUROC remains comparable to SAM2-UNet, indicating similar anomaly discrimination capability.

Compared with SAM2-UNext and SAM-Adapter, the proposed method shows more stable performance across both datasets, especially in segmentation-related metrics. Traditional UNet variants show significantly lower performance, particularly on VisA, indicating limited generalization ability under complex industrial conditions.

#### 4.3.3. Experiments Conclusion

Overall, the experimental results demonstrate that the proposed method achieves consistent and robust performance across different datasets and object categories. This indicates strong generalization ability under varying data distributions.

The superior performance can be attributed to the effective integration of a pre-trained SAM2 backbone with parameter-efficient adaptation, a multi-branch feature extraction strategy, and a hierarchical feature fusion mechanism. These components jointly enhance both global semantic understanding and local detail modeling.

In contrast, baseline methods such as U-Net and SAM-based variants either lack sufficient feature representation capacity or fail to effectively model fine-grained defect structures, resulting in inferior segmentation performance.

These results further validate the effectiveness of leveraging foundation models for industrial defect segmentation and highlight the importance of designing task-specific adaptation modules for domain-specific applications.

#### 4.3.4. Stability Analysis

To assess training stability, as shown in Table 2, we retrained the full HCFNet with five random seeds (0, 7, 42, 1024, 2024) on both MVTec AD and VisA under identical settings. As shown in Table 3, the model exhibits low variance across runs on MVTec AD (mDice 0.80 ± 0.01) and reasonable variance on VisA (mDice 0.64 ± 0.03), confirming that the reported performance is stable and not sensitive to initialization.

### 4.4. Ablation Study

#### 4.4.1. Ablation Settings

To further analyze the effectiveness of each component in the proposed model, we conduct a series of ablation studies in MVTec AD dataset. Specifically, we design the following variants: (a) removing the convolutional auxiliary branch to evaluate the effectiveness of multi-branch feature modeling; (b) replacing the Feature Fusion Modules with a 1 × 1 convolution for simple linear projection, in order to verify the importance of multi-scale feature fusion; (c) removing the Gated Adapter module completely to assess its contribution to parameter-efficient adaptation; (d) replacing the Gated Adapter with a standard adapter (w/o Gating) to verify the effectiveness of the gating mechanism; (e) removing the Laplacian operation from the auxiliary branch (w/o Laplacian) to evaluate its impact on detail enhancement.

All ablation experiments are conducted under the same training settings and hyperparameters as the main experiments (see Section 4.1), ensuring a fair comparison.

#### 4.4.2. Ablation Results and Analysis

The ablation results are summarized in Table 3. It can be observed that removing any component leads to a performance degradation, indicating that each module contributes positively to the final performance.

Specifically, removing the convolutional auxiliary branch (a) results in a decrease in mDice and mIoU, demonstrating that the branch plays an important role in capturing fine-grained details and boundary information.

Replacing the Feature Fusion Modules with a 1 × 1 convolution (b) causes further performance decline, indicating that simple linear projection is insufficient for modeling multi-scale defects.

For the adapter module, we observe a progressive performance trend. Removing the adapter entirely (c) leads to the most significant performance drop (mDice drops to 0.716), demonstrating the necessity of parameter-efficient adaptation. Replacing our Gated Adapter with a standard adapter (d) yields a moderate performance (mDice 0.784), which is lower than our full model (0.802). This comparison validates that the gating mechanism is superior to the standard adapter design, as it effectively modulates feature adaptation.

Furthermore, removing the Laplacian component (e) also results in a performance drop compared to the Raw model, confirming that the Laplacian operation contributes positively to feature representation.

#### 4.4.3. Complexity Analysis

To assess computational efficiency, as shown in Table 4, we report the number of parameters and FLOPs of the proposed model and its ablation variants. The full HCFNet contains 240.5 M parameters, of which only 28.4 M (11.8%) are trainable (the Gated Adapters, auxiliary branch, fusion modules, and decoder), while the SAM2 Hiera-L backbone remains frozen. At a 352 × 352 input, the full model requires 135.0 G FLOPs. Notably, the Gated Adapter and the Laplacian-enhanced auxiliary branch introduce negligible additional parameters and FLOPs relative to the backbone, confirming the efficiency of the proposed design. In addition, we measured inference latency on a single NVIDIA RTX 3090 (batch size 1, 352 × 352 input, averaged over 100 runs): HCFNet achieves 32.18 ms/image (31.1 FPS), confirming that the added Gated Adapter and Laplacian branch introduce negligible overhead and that the model remains suitable for practical deployment.

#### 4.4.4. Per-Category Evaluation

**Figure 9:**
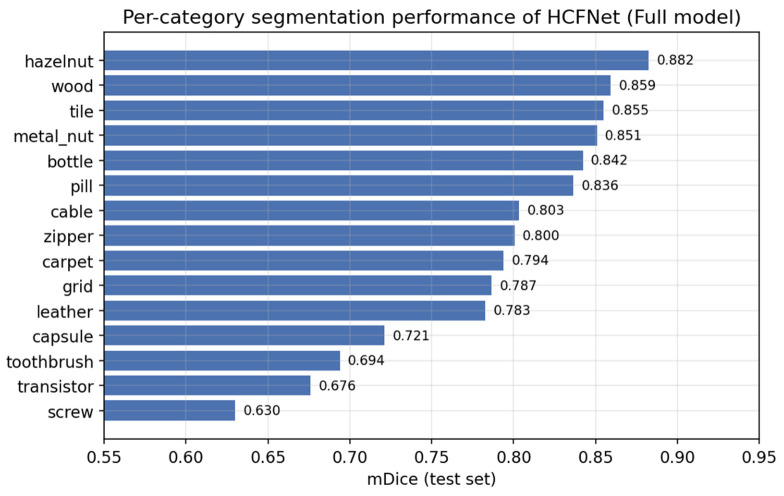
Per-category mDice of HCFNet on the MVTec AD test set.

To provide a more detailed assessment, we evaluate the full model on each of the 15 MVTec AD categories. As shown in Figure 9, HCFNet achieves competitive mDice across all categories (e.g., hazelnut 0.882, wood 0.859, tile 0.855, metal_nut 0.851, bottle 0.842), demonstrating stable performance over diverse defect types and materials.

**Figure 10:**
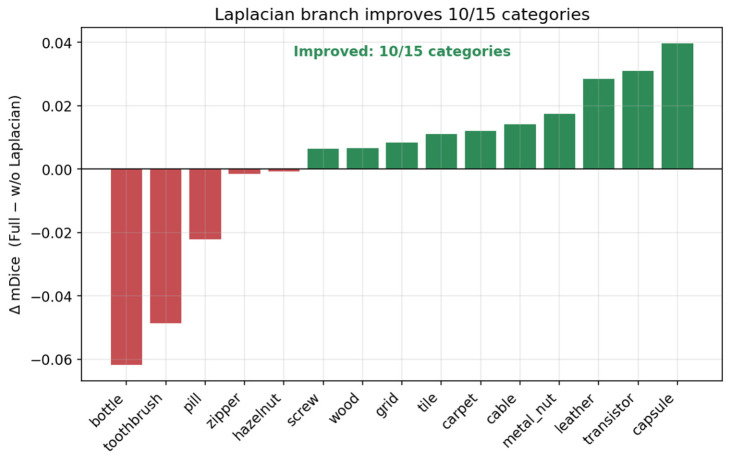
Per-category mDice difference (Full minus w/o Laplacian); green: improved.

We further examine the contribution of the Laplacian-enhanced auxiliary branch at the category level. As shown in Figure 10, compared with the variant without the Laplacian branch, the full model achieves higher mDice on 10 of the 15 categories, with the most pronounced gains on fine-grained and texture defect categories (capsule +0.040, transistor +0.031, leather +0.028), consistent with the design intent of enhancing high-frequency detail perception.

#### 4.4.5. Small-Defect Analysis

Since micro-cracks and tiny defects are a primary motivation of this work, we report a size-stratified evaluation on the MVTec AD test set. Defects are grouped by ground-truth mask area into three equal-frequency bins: small, medium, and large (each approximately 63 images). As shown in Table 5, performance scales monotonically with defect size, while the model still attains competitive segmentation on small defects (mDice 0.723/mIoU 0.592). Together with the per-category gains on fine-grained categories (e.g., capsule +0.040, transistor +0.031, leather +0.028 mDice from the Laplacian branch), this indicates that the frequency-aware design preserves sensitivity to fine and small-scale defects rather than only optimizing for large regions.

**Figure 11:**
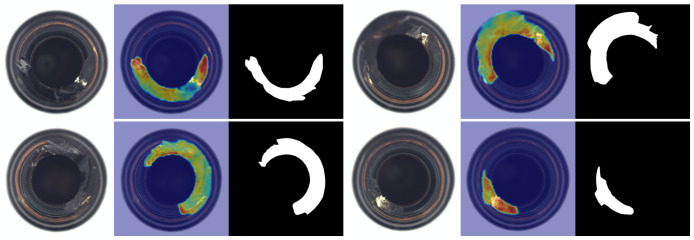
Grad-CAM visualizations on representative MVTec AD defects.

To further examine how the model perceives defects, we visualize its attention using Grad-CAM [49] on representative samples, as shown in Figure 11. The activation consistently concentrates on defect regions and their fine boundaries, including small-scale anomalies, indicating that the frequency-aware auxiliary branch directs the model’s attention toward the high-frequency details that characterize industrial defects.

**Figure 12:**
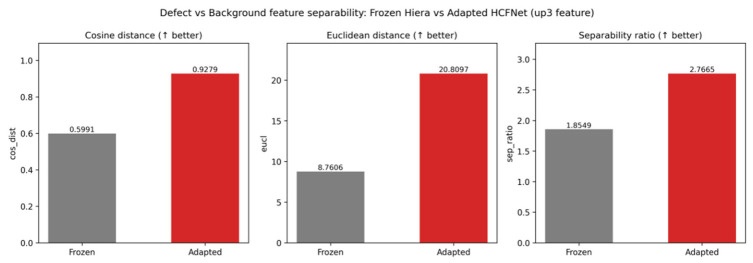
Defect vs. background feature separability with the Gated Adapter disabled (Frozen) and enabled (Adapted): inter-class cosine distance, Euclidean distance, and inter/intra separability ratio.

To directly verify the Gated Adapter’s effect on domain adaptation, we measure defect vs. background feature separability at the segmentation-head input under the same trained weights, with the adapter residual disabled (Frozen) or enabled (Adapted). As shown in Figure 12, enabling the trained adapter raises the inter-class cosine distance from 0.599 to 0.928 (+54.9%) and the inter-/intra-class separability ratio from 1.85 to 2.77 (+49.1%), and roughly doubles the Fisher discriminant ratio (80.9 to 162.2, +100.5%). This confirms that the Gated Adapter substantially increases defect/background discriminability, mitigating the domain shift between the natural-image pretrained backbone and industrial defects.

## 5. Conclusions

In this paper, we propose the Hierarchical Cross-Branch Frequency-Aware Network (HCFNet) to effectively adapt the SAM2 foundation model for industrial defect segmentation. To address the critical challenges of domain shift, local detail insensitivity, and prompt dependency inherent in migrating SAM2 to industrial scenarios, we introduce a dual-branch paradigm that explicitly decouples global semantic modeling from local detail enhancement. By synergizing a Gated Adapter for parameter-efficient domain transfer, a Laplacian-enhanced auxiliary branch for high-frequency feature extraction, and a cross-branch multi-scale fusion strategy, our framework successfully bridges the gap between general visual priors and domain-specific industrial requirements. Extensive experiments demonstrate the competitive defect segmentation performance of HCFNet on representative industrial materials such as steel and ceramics, achieving state-of-the-art performance with an mDice of 80.2% and an mIoU of 69.5% on the MVTec AD dataset. Beyond the quantitative improvements, this work provides a prompt-free approach for deploying foundation models in automated industrial inspection systems. Future work will focus on optimizing computational efficiency and extending this framework to broader real-world manufacturing applications. 

## Figures and Tables

**Table 1:** Quantitative comparison of different methods on the industrial defect segmentation task.

Method	MVTec AD				VisA			
	mDice	mIoU	AUROC	MAE	mDice	mIoU	AUROC	MAE
Ours	0.802	0.695	0.969	0.007	0.645	0.518	0.942	0.004
Unet_30	0.346	0.251	0.929	0.055	0.227	0.154	0.871	0.015
Unet_100	0.635	0.514	0.961	0.017	0.368	0.268	0.875	0.015
SAM2-Unet	0.771	0.657	0.979	0.009	0.625	0.498	0.942	0.005
SAM2-UNext	0.758	0.661	0.926	0.019	0.567	0.460	0.908	0.010
SAM-Adapter	0.749	0.658	0.943	0.015	0.095	0.070	0.886	0.120

**Table 2:** Stability of HCFNet over five random seeds (mean ± std).

Dataset	mDice	mIoU	AUROC	MAE
MVTec AD	0.8028 ± 0.0053	0.6938 ± 0.0071	0.9778 ± 0.0027	0.0087 ± 0.0004
VisA	0.6402 ± 0.0265	0.5147 ± 0.0264	0.9406 ± 0.0098	0.0035 ± 0.0007

**Table 3:** Ablation results for each core component on MVTec AD.

Method	mDice	mIoU	AUROC	Sα	Eφ	MAE
raw	0.802	0.695	0.969	0.872	0.946	0.007
a	0.795	0.685	0.974	0.865	0.938	0.008
b	0.787	0.677	0.978	0.859	0.928	0.009
c	0.716	0.598	0.964	0.818	0.899	0.012
d	0.784	0.672	0.975	0.854	0.928	0.008
e	0.793	0.682	0.980	0.858	0.929	0.008

**Table 4:** Parameters and FLOPs of HCFNet and its ablation variants.

Variant	Params	FLOPs
Full (HCFNet)	240.5 M	135.0 G
Without Adapter	212.6 M	122.8 G

**Table 5:** Size-stratified defect segmentation on MVTec AD.

Defect Size	Images	mDice	mIoU
Small	63	0.7232	0.5920
Medium	64	0.8317	0.7265
Large	63	0.8846	0.8049
Overall	190	0.8133	0.7079

## Data Availability

The data that support the findings of this study are available from the corresponding author upon reasonable request. The benchmark datasets used in this study (MVTec AD and VisA) are publicly accessible via their official repositories: MVTec AD (https://www.mvtec.com/research-teaching/datasets/mvtec-ad, accessed on 1 December 2025) and VisA (https://github.com/amazon-science/spot-diff, accessed on 1 December 2025).

## Abbreviations

SAM	Segment Anything Model
PEFT	Parameter-Efficient Fine-Tuning
HCFNet	Hierarchical Cross-Branch Frequency-Aware Network
RFB	Refined Feature Block
FLOPs	Floating-point Operations
mDice	mean Dice coefficient
SAM2	Segment Anything Model 2
LoRA	Low-Rank Adaptation
Hiera-L	Hierarchical Encoder (Large)
SE	Squeeze-and-Excitation
mIoU	mean Intersection-over-Union
AUROC	Area Under the ROC Curve
